# Obituary: Dominique Job (1947-2022)

**DOI:** 10.3389/fpls.2023.1188766

**Published:** 2023-05-08

**Authors:** Elisabeth Jamet, Marie-Thérèse Esquerré-Tugayé, Karine Gallardo-Guerrero, Norbert Rolland, Michel Zivy, Mélisande Blein-Nicolas, Delphine Vincent, Brigitte Gontero, Loïc Rajjou

**Affiliations:** ^1^ Laboratoire de Recherche en Sciences Végétales, Université de Toulouse, CNRS, UPS, Toulouse INP, Auzeville-Tolosane, France; ^2^ Agroécologie, INRAE, Institut Agro, Univ. Bourgogne, Univ. Bourgogne Franche-Comté, Dijon, France; ^3^ Laboratoire de Physiologie Cellulaire & Végétale, Université Grenoble Alpes, CNRS, INRAE, CEA, IRIG–LPCV, Grenoble, France; ^4^ Université Paris-Saclay, INRAE, CNRS, AgroParisTech, GQE - Le Moulon, PAPPSO, Gif-sur-Yvette, France; ^5^ Agriculture Victoria, AgriBio, Centre for AgriBioscience, Bundoora, VIC, Australia; ^6^ Aix Marseille University, CNRS, UMR7281 Bioénergétique et Ingénierie des Protéines, Marseille, France; ^7^ Université Paris-Saclay, INRAE, AgroParisTech, Institut Jean-Pierre Bourgin (IJPB), Versailles, France

**Keywords:** Dominique Job, obituary and bibliography, plant physiologist, French Academy of Agriculture, proteomics

Dominique Job was a renowned plant physiologist. He was born on June 4^th^ 1947 in Marseille and passed away on October 18^th^ 2022. He was initially trained in Physics and Mathematics. In 1969, he obtained a Master`s degree in Physics at the University of Dakar (Senegal). Subsequently, he joined the Laboratory of Plant Biochemistry (CNRS/Aix-Marseille University, France) where he conducted research on the physicochemical and enzymatic properties of hemeproteins from plants. He primarily used rapid enzyme kinetic approaches such as stopped-flow and temperature-jump relaxation, as well as other spectroscopic methods at very low temperature, which was highly innovative at the time. Dominique was particularly grateful to Prof Douzou for sharing his expertise on this technique during an internship in his lab (Institut de Biologie Physico-Chimique, Paris). This work was the basis of his first article published in European Journal of Biochemistry (Ricard and Job, 1974). In 1970, Dominique was hired by CNRS as a Research Associate and in 1975, he successfully defended his PhD thesis. This research on plant hemeproteins was extended during two postdoctoral fellowships, first at the Department of Chemistry of the University of Edmonton (Canada) in the laboratory of Prof Dunford (1975-1977) and then at the University of Newcastle upon Tyne (UK) in the Radiation and Biophysical Chemistry Laboratory led by Prof Jones (1977).

**Figure:**
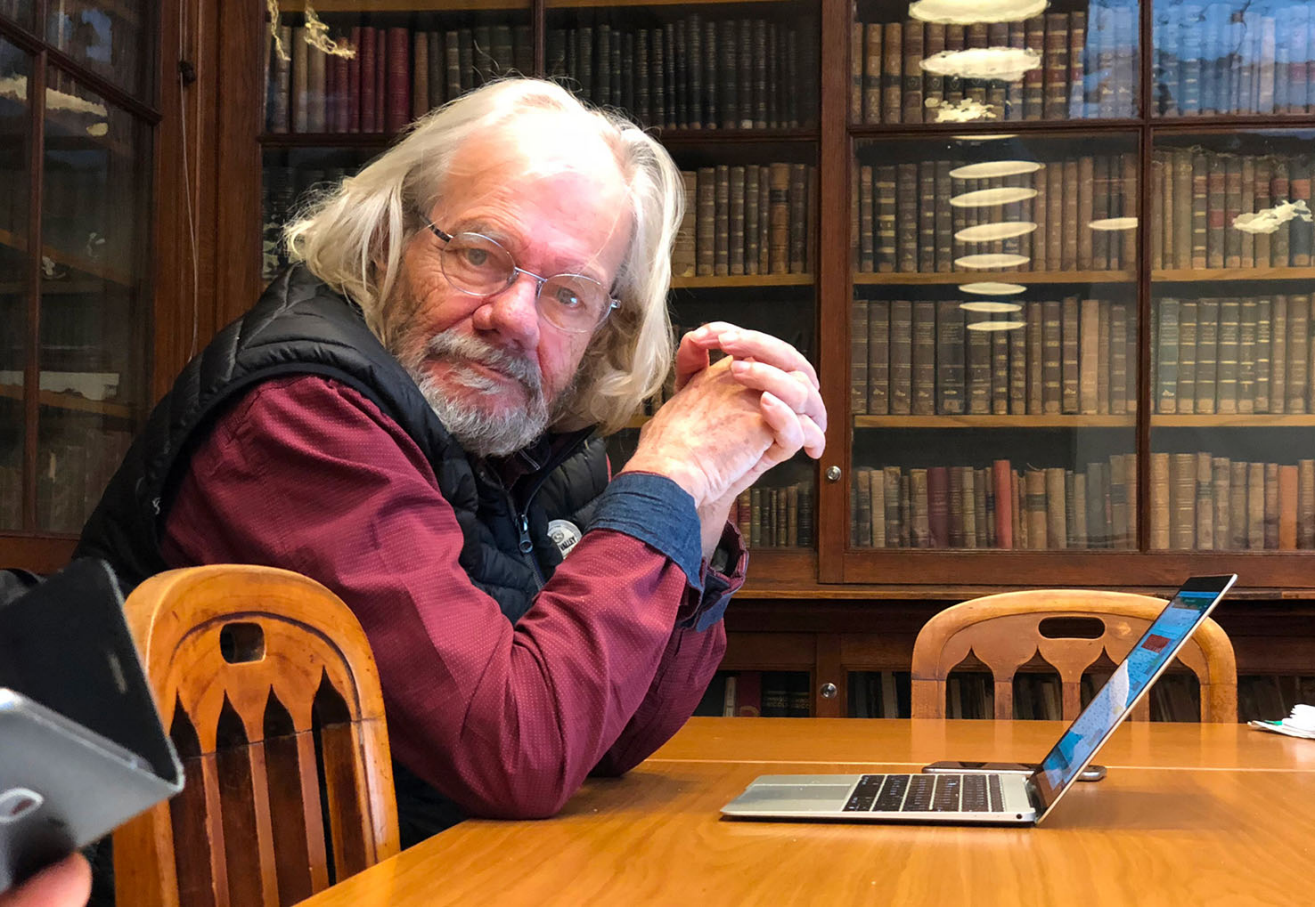
Dominique Job sitting in the library of the French Academy of Agriculture in January 2018.

In 1978, upon returning to Marseille, Dominique started an emerging topic with the study of the mechanisms of transcription in plants. His major discoveries included (i) the elucidation of the mechanisms contributing to the processivity of the RNA polymerase II, (ii) the factors influencing the balance between abortive and productive transcription initiation, (iii) the mechanism of action of α-amanitin (a specific inhibitor of RNA pol II), and (iv) the influence of the sequence and conformation of the DNA template on the velocity and fidelity of transcription.

In 1992, Dominique moved to Lyon to integrate a public-private joint laboratory between CNRS and Rhône-Poulenc that later became CNRS/Bayer CropScience. He initiated a new project focusing on seed biology, while also conducting research on the characterization of the enzymatic pathways responsible for amino acid metabolism (sulfur or branched chain amino acids) and vitamin metabolism, particularly with regards to biotin. In 1999, Dominique was at the forefront of the development of plant proteomics by conducting a comprehensive analysis of the seed proteome of the model plant, *Arabidopsis thaliana*, made possible by the decoding of this first plant genome. Passionate about seed biology and the mechanisms that govern the transition from a dormant to an active metabolic state during germination, he has made a remarkable contribution to the generation of knowledge in this area of science and his articles are widely cited. In particular, a rule was invented in honor of the work achieved in Dominique’s lab. It states that “*The proteins critical for translation must endure desiccation, quiescence, and rehydration in a functional state if the seed is to survive to complete germination*” (Dirk and Downie, 2018). One of Dominique’s latest research interests dealt with *Amborella trichopoda*, the sister of all flowering plants. Dominique’s findings indicate that Amborella 11S globulins share similarities to those found in monocots and eudicots and display specific characteristics of corresponding seed storage proteins in basal angiosperms and gymnosperms. This supports the use of 11S globulins for further phylogenetic studies.

From 2008 to 2014, Dominique served as a Consultant Professor within the Chair of Plant Physiology at AgroParisTech, a leading French institution of higher education in the field of agricultural and environmental sciences as well as food technology. Dominique was widely recognized for his exceptional pedagogy, able to motivate and inspire his students, whether they be in engineering, master, or doctoral programs. Through his clear and concise approach to plant sciences, he was able to explain even the most complex concepts in biology, biochemistry, and genomics in a manner that was both comprehensible and relevant to his audience. His charisma and passion for life science research made him a highly valued professor, awaking interest and creativity in his students. He provided insightful advice to help students navigate their academic and career paths and accelerate their scientific success.

Besides research in the lab, Dominique has exerted a number of important responsibilities as a deputy head or head of the public-private joint laboratory CNRS/Bayer CropScience (UMR CNRS 5240) over the 1993-2010 period. At the national level, from 2000 to 2010, Dominique has been involved in the promotion of plant genomics as the scientific coordinator and manager through the *Génoplante* programme, a French public-private partnership. From 2008 to 2010, Dominique was Chairman of the scientific committee (calls for projects in plant and animal genomics) for the French National Research Agency (ANR). He served as a driving force in connecting basic research issues with practical concerns in the plant breeding industrial sectors. In 2012-2013, he was a scientific delegate for the French Evaluation Agency of Research and Higher Education (HCERES). At the European level, since 2006, Dominique was a member of the strategic advisory board of the German program for plant genomics and biotechnology (GABI, then Plant 2030 - Plant Biotechnology for the Future), and for the European PLANT-KBBE (“PLant Alliance for Novel Technologies - towards implementing the Knowledge-Based Bio-Economy in Europe”). Moreover, since 2008, Dominique has been working toward the establishment of an International Plant Proteomics Organization (INPPO), of which he was the first President (2008-2014) and which currently counts more than 700 members from over 60 countries.

After his retirement, Dominique remained an active scientist. He was nominated as an Emeritus CNRS Research Director in 2012 and he also became a talented Academician. Since his entry at the French Academy of Agriculture as corresponding (2004), then full member (2013), he never failed to promote the contribution of basic knowledge to the advancement of agriculture. Upon his arrival as secretary leader of the *Life science* section (2013-2017), and ever since, he readily innovated by systematically organizing a series of seminars by invited speakers, the so-called “*At the frontiers of…*” series. His management of national and European programs combined to his endless curiosity afforded Dominique a broad overview of new trends in plant science and beyond, which helped recruiting members with novel skills, for instance in the area of synthetic biology. As a defender of transdisciplinary approaches, Dominique set up several meetings coordinated with other sections, and Academies (Science, Technologies). Topics recently covered and in preparation were *Biodiversity* (2021), a theme very close to his heart (https://www.academie-agriculture.fr/actualites/academie/colloque/academie/distribution-et-dynamique-de-la-biodiversite); e*DNA from paleogenomics to nowadays landscapes* (planned for November 2023); and *Mathematics and Biology* (in preparation). As an active participant of the Academy, he contributed to working groups, namely “*One health”* through a chapter inspired by the Plant Health colloquium that he co-organized in Toulouse in November 2021 (https://www.youtube.com/watch?v=t5rt9-vU0H0); and “*Agriculture Biologique*” (https://www.academie-agriculture.fr/publications/articles/les-donnees-omiques-permettent-de-distinguer-les-produits-de-lagriculture). Over the years, Dominique has consistently taken part in the committees responsible for supporting and awarding the research of both senior and junior scientists in the field of agriculture.

Dominique became an authority on so many plant science topics that his opinion as an editor was highly valued. He was a member of the Editorial Board of Molecular & Cellular Proteomics, Seed Science Research, and Scientific Reports, Associate Editor of Frontiers in Plant Science, and Associate Chief Editor of Proteomes. This long list reflects his multiple scientific interests and technical expertise; it also illustrates his advocacy in promoting post-genomics, particularly proteomics. In this last aspect, Dominique also actively participated to the animation of the French Green Proteome network (https://www6.inrae.fr/proteome-vert), where he communicated his enthusiasm to many students and researchers. In 2007, he played an important role in setting up the French seed biology network. Since then, this network has organized a national symposium every two years, which has become a major event for academic researchers and seed industry professionals to come together, exchange information and cooperate. He was a permanent member of the scientific council of this network. As an editor, Dominique allowed the reporting of numerous high-quality peer-reviewed articles that filled a scientific knowledge gap, advanced biotechnologies, and reviewed the state-of-the-art of the many biological themes he specialized in. From 2011 to 2022, Dominique edited seven Frontiers in Plant Science’s Research Topics, aiming at unifying the most influential researchers, recent findings and cutting-edge progress in dynamic research areas pertaining to plant-microbe interactions, plant infection and defense, cereal and seed biology, as well as agricultural biotechnology. Such far-reaching editorial activities served Dominique’s future thinking process by making him aware of areas that were actively researched and on the verge of publication. Dominique’s contribution as an editor was immense; it helped boost the scientific fields he was passionate about, in which plants, seeds and proteomics research featured prominently.

Dominique could absorb an impressive amount of knowledge on any topic he set his mind to in a record time. He had a steely, incisive mind, and an unlimited curiosity. He was an open, free-minded, enthusiastic and visionary person who dedicated his life to Science. He was always willing to impart his wisdom faithfully and clearly to others, particularly emerging biologists, and discuss his findings with his many peers. Throughout his extensive scientific career as a researcher, an author, a reviewer, an editor, as well as a prized member of several committees and associations, Dominique kept abreast of countless aspects of biology, not only on plant but also human, animal and microbial sciences. He was constantly seeking out the newest discoveries, latest technologies and best strategies for practical applications to progress crop science and support an environmentally-friendly and sustainable agriculture. With an h-index of 60, he co-authored 218 publications with 14,268 citations (Google scholar on February 23^rd^ 2023), many of them with his lifelong collaborator Claudette Job. In 2023, he is ranked 267 among the French scientists and 7,177 at the world level (https://research.com/u/dominique-job). His research was published in reputable and high impact factor journals such as Science, Proceedings of the National Academy of Science of the USA, and The Plant Cell, to name just a few. In 2019, he was awarded as a *Chevalier de l’ordre du Mérite agricole* for his entire career.

Dominique was very helpful and encouraging and spent a lot of his time sponsoring, supporting and advising young scientists more like a mentor than a supervisor. He had an exceptional ability to communicate his scientific knowledge. Many of his students followed in his footsteps and became scientists; they feel privileged to have contributed to his research. Inside and outside of the laboratory, he was kind, social, generous, and faithful. He will also remain in our memories as a remarkable Academician, a gentleman. Dominique will be immensely missed by his wife Claudette, his son Stéphane, his daughter Sandrine, his grand-children Ethan, Basile and Marius, and his many scientific friends and colleagues from around the world. The furrow he dug for them and for all of us will remain an invaluable guideline, forever.

## Job's rule

Dirk, L. M. A., and Downie, B. (2018). An examination of Job’s rule: protection and repair of the proteins of the translational apparatus in seeds. Seed Sci. Res. 28, 168–181. doi:10.1017/S0960258518000284

## Author contributions

All authors contributed to the article and approved the submitted version.
